# Core regulon of the global anaerobic regulator Anr targets central metabolism functions in *Pseudomonas* species

**DOI:** 10.1038/s41598-019-45541-0

**Published:** 2019-06-21

**Authors:** Paula M. Tribelli, Adela M. Lujan, Agustín Pardo, José G. Ibarra, Darío Fernández Do Porto, Andrea Smania, Nancy I. López

**Affiliations:** 1 IQUIBICEN, CONICET, Universidad de Buenos Aires, Buenos Aires, Argentina; 2 Departamento de Química Biológica, Facultad de Ciencias Exactas y Naturales, Universidad de Buenos Aires, Buenos Aires, Argentina; 3 Universidad Nacional de Córdoba, Facultad de Ciencias Químicas, Departamento de Química Biológica Ranwel Caputto, Córdoba, Argentina; 4 CONICET, Centro de Investigaciones en Química Biológica de Córdoba (CIQUIBIC), Córdoba, Argentina; 5 Instituto de Cálculo, Facultad de Ciencias Exactas y Naturales, UBA, Buenos Aires, Argentina

**Keywords:** Gene regulatory networks, Bacterial genomics

## Abstract

A comparative genome analysis of the global anaerobic regulator Anr regulon in five species of *Pseudomonas* with different life style was performed. Expression of this regulator was detected in all analyzed *Pseudomonas*. The predicted Anr regulon (pan-regulon) consisted of 253 genes. However, only 11 Anr-boxes located upstream of *qor/hemF, hemN, cioA*/PA3931, *azu*, *rpsL, gltP*, orthologous to PA2867, *cspD*, *tyrZ, slyD* and *oprG*, were common to all species. Whole genome *in silico* prediction of metabolic pathways identified genes belonging to heme biosynthesis, cytochromes and Entner-Doudoroff pathway as members of Anr regulon in all strains. Extending genome analysis to 28 *Pseudomonas* spp. spanning all phylogenetic groups showed Anr-boxes conservation in genes related to these functions. When present, genes related to anaerobic metabolism were predicted to hold Anr-boxes. Focused on the genomes of eight *P. aeruginosa* isolates of diverse origins, we observed a conserved regulon, sharing nearly 80% of the genes, indicating its key role in this opportunistic pathogen. The results suggest that the core Anr regulon comprises genes involved in central metabolism and aerobic electron transport chain, whereas those genes related to anaerobic metabolism and other functions constitute the accessory Anr-regulon, thereby differentially contributing to the ecological fitness of each *Pseudomonas* species.

## Introduction

*Pseudomonas* species are metabolically versatile bacteria that can thrive in diverse environmental conditions. Aerobic respiration is the privileged energy generation mechanism^1^ but several members of this genus are able to develop in a gradient of oxygen conditions, from aerobiosis to anaerobiosis, using different mechanisms for obtaining energy. The Anr regulator (for anaerobic regulation of arginine deiminase and nitrate reduction) is a key factor involved in metabolic plasticity regarding oxygen availability in *Pseudomonas* species^2,3^. Anr is homologous to *Escherichia coli* Fnr (fumarate and nitrate reductase regulator)^4,5^. Its ability to sense oxygen levels lies in either a [4Fe–4S]^2+^or a [2Fe–2S]^2+^cluster bound to four Cys residues. In *E. coli*, under low oxygen conditions, the reduced dimeric active form of Fnr binds to conserved DNA binding sites in promoters (Fnr-box) and regulates target genes transcription^6^. Besides the typical metabolic processes related to energy generation such as denitrification, arginine and pyruvate fermentation, Anr also controls a variety of metabolic functions as redox state maintenance, fimbria and cytochrome biosynthesis, secretion type III system, oxidative stress resistance and quorum sensing cascades^3,4,7–10^.

Most of the knowledge about Anr modulated functions is based on *Pseudomonas aeruginosa*, the type species of the genus. In this bacterium, two CRP/FNR transcription factors, Anr and Dnr regulators, are involved in the response to oxygen availability and the presence of N-oxides^11,12^. By contrast, the knowledge of the regulatory role of Anr in other *Pseudomonas* species is still limited. In *P. stutzeri*, a facultative anaerobic bacterium, four Fnr-like regulators control the expression of denitrification in response to oxygen levels^13^. In *P. putida*, an obligate aerobic soil bacterium used as cell factory^14^, Anr has an important role on expression level and coordination of the different terminal oxidases belonging to the branched aerobic respiratory chain in response to oxygen availability^2^. The role of Anr in different cellular process including polyhydroxybutyrate metabolism, redox state, oxidative stress resistance and biofilm development has been reported in the Antarctic bacterium *P. extremaustralis* under low oxygen conditions^15–17^. In the strictly aerobic *P. protegens* CHA0 (formerly *P. fluorescens* CHA0), Anr is required for hydrogen cyanide synthesis, a compound that contributes to biocontrol abilities of this strain^18^.

Here we confirmed the expression of Anr mRNA and protein in 5 model *Pseudomonas* with different ecological niches, biotechnological applications and impact in human activities followed by a comparative *in silico* analysis of the Anr regulon. Bioinformatic analyses together with whole genome metabolic network reconstruction were used to determine the core and accessory regulon in these five bacteria. Inter and intraspecific variability of the functions controlled by this master regulator was examined by extending the analysis to other *Pseudomonas* spp. Our results predicted a set of core Anr-controlled genes related to major factors and pathways central to energy generation, in both obligate aerobes and facultative anaerobes, whereas a large set of genes showed species-to-species variation with respect to the presence of Anr-boxes, probably as a reflection of their physiological, biochemical and ecological properties.

## Results

### Characterization of Anr regulator in model *Pseudomonas* species

In order to examine the interspecific diversity of the Anr regulon, we selected five representative species of the *Pseudomonas* genus, including environmental and human and plant pathogens strains. The environmental strains comprised two soil isolates (*P. putida* KT2440 and *P. protegens* Pf-5) and the extremophile bacteria *P. extremaustralis* 14-3b isolated from a temporary water pond in Antarctica (Table 1), whereas pathogens included *P. aeruginosa* PAO1, an opportunistic human pathogen, and *P. syringae* pv. *syringae* B728a, a plant pathogen. Genome sizes ranged from 6.09 to 7.07 Mb and G + C content from 59.2 to 66.6% (Table 1). The anaerobic global regulator *anr* gene was found in *P. syringae* pv. *syringae* B728a and *P. protegens* Pf-5 genomes in addition to *P. extremaustralis* 14-3b, *P. putida* KT2440 and *P. aeruginosa* PAO1, in which it has been reported before^2,5,16^. The genomic region where *anr* is located showed similarity and high degree of synteny in all *Pseudomonas* species (Supplementary Fig. S1), with the *apt* gene, encoding adenine phosphoribosyl transferase, located always upstream of *anr*. However, the *hemN* gene, encoding coproporphyrinogen III oxidase, is flanking downstream *anr* in *P. extremaustralis* and *P. putida* KT2440, whereas an orthologous to *pemB*, encoding a T3SS effector^19^, resides between *anr* and *hemN* in the remaining analyzed species (Supplementary Fig. S1). Anr sequence was highly conserved among the different species with an average 93.0% of amino acid similarity and 82.7% of identity. All the sequences harboured the four cysteine residues (Cys) that comprise the sensor oxygen domain and the HTH domain necessary for DNA regulation (Supplementary Fig. S2). Phylogenetic tree of Anr deduced protein sequences showed that the sequences clustered according to the species groups reported before^20^, suggesting that *anr* belongs to the core genome of *Pseudomon*as species (Supplementary Fig. S3).

**Table 1:** Characteristics of selected *Pseudomonas* species.

Strain	Source	Relevant characteristic	Genome Size (bp)	Genome Accession #	Protein #	%G + C
*P. aeruginosa* PAO1	Wound isolate	Human opportunistic pathogen	6,264,404	AE004091.2	5,572	66.6
*P. extremaustralis* 14-3b	Temporary pond in Antarctica	Extremophile	6,586,240	AHIP00000000.1	5,870	60.7
*P. protegens* Pf-5	Rhizosphere of cotton seedlings	Biocontrol agent	7,074,893	CP000076.1	6,250	63.3
*P. putida* KT2440	Derived from toluene-degrading soil isolate, mt-2	Soil bacterium, GRAS	6,181,873	AE015451.1	5,564	61.5
*P. syringae* pv *syringae* B728a	Snap bean leaflet in Wisconsin	Plant pathogen	6,093,698	CP000075.1	5,089	59.2

Genome information included for comparison, was obtained from National Center for Biotechnology Information (www.ncbi.nlm.nih.gov). G + C, guanine plus adenosine mols percent. GRAS: generally recognized as safe.

**Figure 1:**
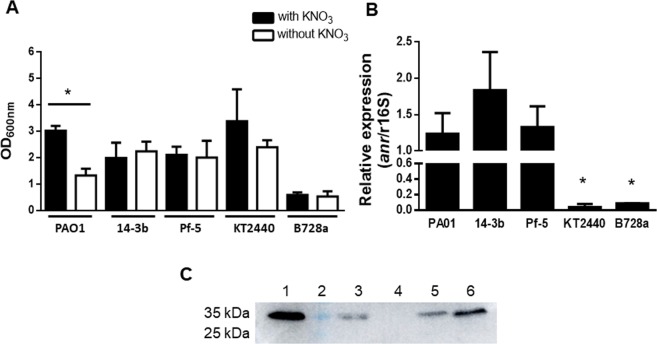
Characterization of microaerobic growth and Anr expression in *Pseudomonas* species. (**A**) Growth under low oxygen conditions with and without KNO_3_. (**B**) Expression of *anr* measured by Real time qPCR. (**C**) Western Blot analysis of Anr. Lines: 1. Purified recombinant Anr protein of *P. extremaustralis* used as positive control 2: Marker PageRuler prestained protein ladder (Thermo scientific); 3: *P. extremaustralis*; 4: *anr* mutant of *P. extremaustralis*^16^ used as negative control. 5: *P. syringae* B728a: 6: *P. putida* KT2440. Original Western-blot assay with different exposure times used to build C are shown in Fig. S4. In A: microaerobic cultures were carried out in LB medium supplemented or not with KNO_3_ in sealed bottles with 1:2 medium to flask volume ratio and low agitation (50 rpm), incubated for 30 h. In B and C: cultures were performed with KNO_3_. Bars represent mean ± SE. *: indicate significant differences P < 0.05. *P. aeruginosa* PAO1 (PAO1); *P. protegens* Pf-5 (Pf-5); *P. putida* KT2440 (KT2440), *P. extremaustralis* 14-3b (14-3b) and *P. syringae* pv. *syringae* B728a (B728a).

Subsequently, microaerobic growth in presence and absence of nitrate was analyzed in the five *Pseudomona*s spp. All of them were capable to grow under both conditions (Fig. 1A). No significant differences in microaerobic growth with or without nitrate were observed in 14-3b, Pf-5, KT2440 and B728a (t-test, P > 0.05). However, PAO1 showed significant differences that can be attributed to its capability to efficiently use nitrate through a complete denitrification process (t-test, P > 0.05, Fig. 1A). Although *P. extremaustralis* 14-3b is also able to perform nitrate reduction, it did not show a significant increase in growth at the assayed times. *P. syringae* pv. *syringae* B728a presented the lowest growth level while the other analyzed strains reached OD_600nm_ values between 2 and 3 after 30 h of incubation (P < 0.05 one-way Anova-test; Fig. 1A). In addition, expression of *anr* gene was observed in the five species under microaerobic conditions (Fig. 1B), although significant differences in the expression among them were observed. *P. putida* KT2440 and *P. syringae* B728a showed the lowest values for *anr* expression (P < 0.05 one-way Anova-test; Fig. 1B). However, and for first time, native Anr proteins were detected in both species by western blot assays under microaerobic conditions, using an anti-Anr antibody designed based on amino acid sequence of *P. extremaustralis* (Fig. 1C). Overall results showed high conservation of *anr* and its expression in all chosen *Pseudomonas* species.

### *In silico* comparison of Anr regulon in *Pseudomonas* species

**Figure 2:**
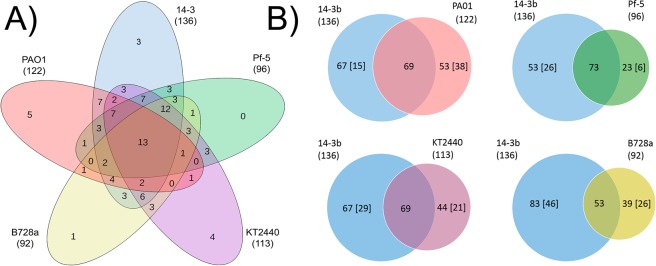
Venn diagram showing the number of genes containing Anr-boxes. (**A**) Comparison of Anr-boxes located upstream of conserved genes present in all *Pseudomonas* species. (**B**) Venn diagram showing the number of genes with predicted Anr box shared between *P. extremaustralis* 14-3b and the other strains. Areas within the pairwise Venn diagrams are drawn to scale and the total number of genes in each species is indicated in parentheses. The numbers in brackets represent unique genes (absent from the other genome). *P. extremaustralis* 14-3b (14-3b); *P. aeruginosa* PA01 (PA01); *P. protegens* Pf-5 (Pf-5); *P. putida* KT2440 (KT2440); *P. syringae pv. syringae* B728a (B728a).

Once *anr* expression was verified in the five *Pseudomonas* model species, an exhaustive genome analysis was performed in order to detect putative Anr-consensus binding sequences (Anr-box) using the PRODORIC software. The complete list of the putative Anr target genes was summarized in Supplementary Table S1. The comparison of the Anr regulons in all species showed the presence of a relatively similar number of putative Anr-boxes in *P. extremaustralis* (136) and *P. aeruginosa* PAO1 (122), followed by *P. putida* KT2440 (113), *P. protegens* Pf-5 (96) and *P. syringae* B728a (92) (Supplementary Table S1), being boxes present in: orthologous genes present in all species, genes not shared among all species, and strain-specific genes. The predicted *Pseudomonas* Anr regulon (pan-regulon) consisted of 253 genes that contains Anr-box in at least one species and covered a wide range of functional categories (Supplementary Fig. S5). Among them, we focused on a subset of 105 orthologous genes that were present in all species although not in all of them contained Anr-boxes. The comparison of these 105 genes revealed a core Anr regulon shared in all analysed species, which putatively could regulate 13 genes, as the Anr-boxes were located at adequate distance in the intergenic region of *rpsL*, *hemN, azu, gltP*, orthologous to PA2867, encoding a protein related to chemotaxis, *cspD*, *sylD*, *tyrZ* and *oprG*, but also upstream of genes transcribed in opposite direction: *qor/hemF* and *cioA*/PA3931 (Fig. 2A, Supplementary Table S1). On the other hand, the analysis showed that *P. extremaustralis* had 3 unique Anr-boxes regulating *groEL*, *ftsB* and *hisG* (Fig. 2A). In *P. aeruginosa* PAO1, 5 unique Anr-boxes were found upstream of PA0864, PA1558, *yhbH*, PA4902 and *yieO*, whereas in *P. putida* KT2440, 4 unique Anr-boxes were identified upstream of *glxR*, *capB*, *dcd* and PP1135 and only 1 for *P. syringae* B728a located upstream of Psyr_3920 (*rpmE2*) (Fig. 2A). No unique Anr-boxes were found for *P. protegens* Pf-5. When non-pathogenic model species (*P. extremaustralis* 14-3b, *P. protegens* Pf-5 and *P. putida* KT2440) were compared, Anr-boxes were identified in the promoter zone of *algZ, hemC*, *dnaK*, PP2161, *ppx*, PP0175 and *pepN*, that can be grouped into chaperone, hypothetical protein and regulator protein functions (Fig. 2A, Supplementary Fig. S5). Surprisingly, we could find only one Anr-box shared between the pathogenic species *P. aeruginosa* PAO1 and *P. syringae* B728a upstream of the *radA* gene (Fig. 2A), encoding a DNA repair protein previously related to antibiotic resistance^21^.

As an approach to validate the prediction of Anr controlled genes, experimental data available in GEO database^22^ or from literature were analyzed. Interestingly, in *P. aeruginosa* the core Anr-controlled genes, *hemN*, *azu*, PA2867 and *gltP* were upregulated in anaerobiosis, whereas *rpsL* and *tyrZ* were found downregulated. Furthermore, in PAO1 *anr* deletion mutants the *azu*, *hemN*, *hemF* and *oprG* genes presented lower expression respect to the wild type strain^3,10^ whereas *cioA* showed higher expression in the mutant^10^. In *P. extremaustralis*, 5 of the predicted core Anr-controlled genes showed differential expression under microaerobic conditions, with *azu*, *hemN*, orthologous to PA2867 and *gltP* being upregulated and *cioA* downregulated; accordingly this gene showed increased expression in an *anr* mutant in comparison with the wild type strain, suggesting that Anr acts as repressor of *cio* genes^15,23^. Additionally, in *P. putida* KT2440 the *cioA* was reported to be repressed by Anr^2^ and evidences regarding Anr dependent upregulation of *azu* and *oprG* were also found^24^.

### Anr regulon pairwise comparison’s between *P. extremaustralis* and the other *Pseudomonas* species

To analyze variations in Anr regulated genes from species to species, we performed comparisons between *P. extremaustralis* 14-3b regulon and the regulons of the other *Pseudomonas*. As mentioned, this extremophile species showed the largest set of putatively regulated genes (136) followed by *P. aeruginosa* PAO1 with 122, with which it shared 69 putatively Anr-regulated genes (Fig. 2B). Similar overlap of regulated genes was observed when Anr regulon of *P. extremaustralis* 14-3b was compared with *P. protegens* Pf-5 and *P. putida* KT2440 regulons sharing 73 and 69 genes, respectively (Fig. 2B). *P. syringae* B728a presented the smallest Anr-controlled regulon with 92 genes (Fig. 2B) and shared only 53 genes with *P. extremaustralis* 14-3b.

The higher relatedness between regulons belonging to *P. aeruginosa* PAO1 and *P. extremaustralis* seem to be associated to the presence of several anaerobic metabolic functions (Supplementary Table S1, Fig. S6), as both species can use nitrate as electron acceptor and can perform arginine and pyruvate fermentation under low oxygen conditions^4,8,25^. In fact, a shared set of genes was related to anaerobic metabolism like *ack*, *dnr*, *nosR*, *narK1*, *narX*, *norC*, *arcD*, and oxygen high affinity cytochromes (Supplementary Table S1, Fig. S6). Moreover, Anr-boxes were also detected in several genes important for environmental adaptability and to cope with stressful conditions like *pilG*, related to biofilm formation, the catalase *katA*, *aer*, related to aerotaxis, and *alkB*, a gene that was found to be involved in microaerobic degradation of alkanes in *P. extremaustralis*^23^. Remarkable, *hcnA*, one of the first targets of Anr regulation in *P. aeruginosa*^26^ was not shared with *P. extremaustralis* as hydrogen cyanide synthesis genes are absent in this last bacterium (Supplementary Table S1, Fig. S6). However, boxes on *hcnA* were also predicted for the plant associated bacteria *P. protegens* Pf-5 and *P. syringae* B728a (Supplementary Table S1). Interestingly, as a strain-specific function, *P. extremaustralis* 14-3b showed the presence of Anr-boxes in *phbR*/*phbC*, related to polyhydroxybutyrate (PHB) metabolism that were previously analyzed in an *anr* mutant^16^. This bacterium presents two different clusters for polyhydroxyalkanoates biosynthesis one for polyhydroxybutyrate (PHB), a short length PHA, and another for medium chain length polyhydroxyalkanoate (mclPHA)^27,28^. Interestingly, in *P. extremaustralis* Anr-box was found only in the promotor zone of the PHB related genes, but not upstream the mclPHA, encoding the operon typical of *Pseudomonas* species. In concordance, boxes in genes related to mclPHA were not found in *P. aeruginosa*.

### Prediction of Anr regulation on the *Pseudomonas* metabolic network

According to our results, the core Anr-controlled gene set comprises only around 10% of the Anr regulon from each of the five analyzed species. However, Anr could control the expression of different genes belonging to the same metabolic pathway leading to the whole regulation of a particular metabolic route.

**Figure 3:**
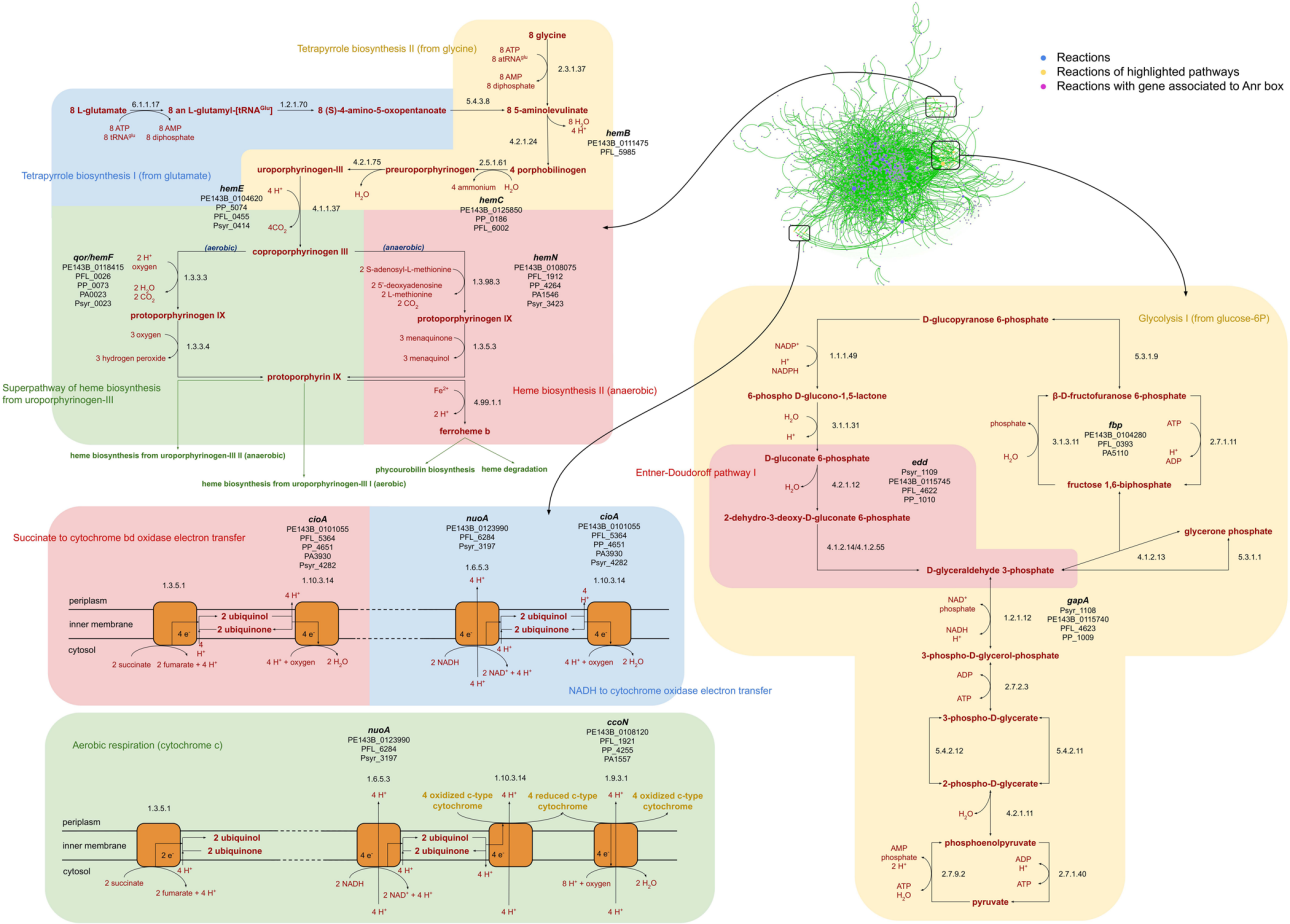
Metabolic network of *Pseudomonas* species. Each node represents a predicted reaction in the *Pseudomonas* metabolism. Heme biosynthesis, Entner Doudoroff pathway and cytochromes are shown in detail.

To test this hypothesis, we performed an *in silico* prediction of metabolic networks (MN) of *P. extremaustralis* using genome information as was described in the methods section and the metabolic reactions containing boxes were identified for all the species. The comparative analysis revealed that, the superpathway of heme biosynthesis, the electron transport chain and the Entner-Doudoroff pathway (EDP) involved different genes in each species that presented Anr-boxes, suggesting that these metabolic pathways are Anr dependent (Fig. 3).

Heme biosynthesis is a key process that provides this component for different cellular proteins^29^. In addition to *qor*/*hemF* and *hemN* that were identified as belonging to the Anr core regulon, *hemE* showed boxes in four of the five model species being absent in *P. aeruginosa* PAO1, *hemC* presented Anr boxes in 3 of the 5 analyzed species (*P. extremaustralis* 14-3b, *P. putida* KT2440, *P. protegens* Pf-5) while *hemB* showed Anr box in 2 of them (*P. extremaustralis* 14-3b and *P. protegens* Pf-5). *P. extremaustralis* showed putative Anr binding sites in all the mentioned genes, suggesting a redundant and a high regulation of this pathway. Reactions predicted as Anr controlled in heme biosynthesis pathway showed different normalized betweenness centrality with values ranging from 0.004 for *hemF* to 0.074 for *hemC* (Fig. 3).

EDP converts glucose into pyruvate with the production of NADH^30^. Although absent in the Anr-core, the presence of Anr-boxes in different genes belonging to EDP, such as *fbp* (fructose 1,6 biphosphate), *gap* (glyceraldehyde 3-phosphate dehydrogenase) or *edd* (phosphogluconate dehydratase) (Fig. 3) in the five *Pseudomonas* species suggests that regulation of these functions by Anr is spread among this genus. EDP and glycolysis represent fundamental metabolic pathways and include one of the most central reactions in the whole metabolism catalyzed by pyruvate kinase (0.73 normalized betweenness centrality), a key node representing a very interconnected reaction (Fig. 3). Reactions predicted as Anr controlled, showed intermediate centrality with the higher value for the reaction catalyzed by *edd* product (0.053 normalized betweenness centrality) in comparison with those catalyze by the enzymes encoded by *fbp* and *gap* (0.028 and 0.024 normalized betweenness centrality, respectively).

Regarding the electron transport chain, besides *cioA* that belongs to the core Anr regulon, *nuoA* presented boxes in 3 species (Fig. 3). The cbb3-type cytochrome c oxidases, cbb3-1 and cbb3-2, encoded by the gene clusters *ccoN1O1Q1P1* and *ccoN2O2Q2P2* were studied in *P. aeruginosa*, as well as in *P. putida* and *P. extremaustralis*. However, *P. putida* cbb3-1 corresponds to cbb3-2 of *P. aeruginosa*^2^. Then, to avoid confusion, in Fig. 3 we decided to use *ccoN* (without number) to include both designations. Boxes were detected in *ccoN* for all species, with the exception of *P. syringae* (Fig. 3). For *P. extremaustralis*, Anr boxes were observed in the promoter zones of the three genes (*cioA*, *nuoA* and *ccoN*). All the predicted Anr controlled reactions involving cytochromes presented similar normalized betweenness centrality showing values around 0.1 (0.129, 0.100, and 0.089 for *ccoN*, *cioA* and *nuoA*, respectively).

### Interspecific Anr regulon among *Pseudomonas* species

**Figure 4:**
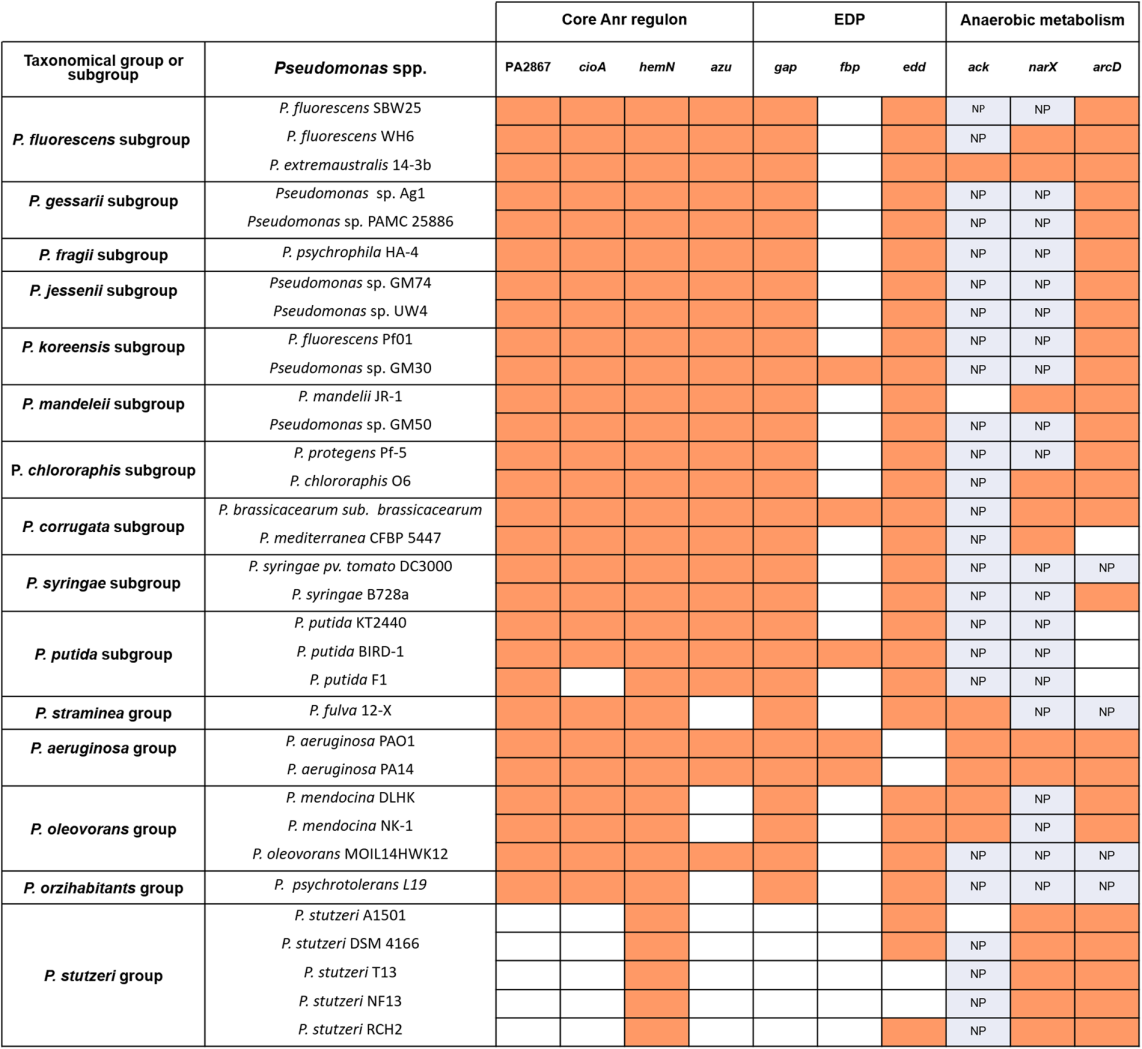
Extended analysis of Anr putative common conserved target genes among 28 *Pseudomonas* species belonging to different phylogenetic groups or subgroups. Orange color indicates presence of Anr box in the promoter region of the gene and white color indicates absence of Anr box. NP: gene not present.

Our integrative Anr regulon analysis allowed the identification of both core Anr-controlled genes and core Anr-controlled pathways in the five representative *Pseudomonas* species. To better characterize core targets of Anr-regulation *in silico* analyses were extended to 28 *Pseudomonas* species belonging to different taxonomical groups or subgroups described before^20^. We searched for Anr-boxes in the promoter zone of genes belonging to the core regulon (orthologous to PA2867, *hemN*, *azu* and *cioA*) and, based on the metabolic network analysis, the presence of Anr-boxes upstream of genes identified in EDP *(gap, edd, fbp)* was also analyzed. All of analyzed species presented Anr-box like sequences upstream of *hemN* reinforcing the importance of heme biosynthesis as key target of this regulator and recognizing this gene as a core target of Anr regulation (Fig. 4). Regarding core Anr regulon genes involved in electron transport chain (*cioA* and *azu*) Anr-boxes were predicted in the promoter zone of both genes in most of the analyzed species (19/28), but were absent in species belonging to *P. stutzeri* subgroup (Fig. 4). Importantly, although *cioA* was not predicted as Anr-regulated in this subgroup, it was reported that the Anr-like regulator, *fnrA*, controls the cytochrome *ccoN* in *P. stutzeri*^13^, reinforcing the idea that core regulation may be focused in the pathway and not in individual genes. It has been also reported that, unlike *P. aeruginosa* PAO1, in *P. putida* KT2440 the cytochrome *cyoA* is controlled by Anr^2^. Since only those Anr boxes located up to 300 bp upstream of the ATG translation initiation codon (see Material and Methods section) were considered, and the Anr box for *cyoA* in *P. putida* KT2440 was located outside (upstream) of this window was not considered in our analysis. However, this report also gives support to the idea that electron transport chain is an important target for Anr regulation. Anr-boxes were detected upstream the orthologous to PA2867 related to chemotaxis in most of the species except in *P. stutzeri* subgroup (Fig. 4). In addition, 26 of the 28 analyzed species presented at least one Anr-box like sequence in *gap or edd* involved in EDP (Fig. 4). Only 2 *P. stutzeri* strains did not show Anr-box in *gap* or *edd*. Remarkable, an Anr box upstream *fbp* promoter zone was only presented in 5 of the 28 analyzed *Pseudomonas* (Fig. 4), suggesting that regulation in this target gene could not be generalized, in concordance with the metabolic network analysis, in which only 3 of the five model species had Anr-boxes in this gene (Fig. 3).

As mentioned, Anr was traditionally related to denitrification, pyruvate and L-arginine fermentation and some cytochromes genes^26^. According to our prediction genes related to these functions that presented Anr-boxes in *P. extremaustralis* and *P. aeruginosa* PAO1 represent species-variable sets of Anr-controlled genes (accessory genes). Then, three genes belonging to anaerobic metabolic pathways were chosen for extending the analysis to the selected 28 species: *ack* (pyruvate fermentation), *arcD* (arginine–ornithine transporter for arginine fermentation) and *narX* (nitrate sensor). *Pseudomonas* spp. that presented genes related with nitrate metabolism (only 11/28, Supplementary Fig. S7) always showed an Anr-box upstream the ATG of *narX* gene (Fig. 4). Similarly, a search for arginine fermentation genes showed that they were present in 24 of the 28 analyzed species (Supplementary Fig. S7). In the case of *arcD*, an Anr-box like upstream ATG was present in 20 species. Only in *P. putida* subgroup and *P. mediterranea* CFBP 5447 the Anr box was absent (Fig. 4). Anr boxes upstream of *ack* were only observed in 4 species including *P. fulva* and those belonging to *P. aeruginosa* and *P. oleovorans* subgroups (Fig. 4).

### Anr regulon in environmental and clinical strains of *P. aeruginosa*

The opportunistic pathogen *P. aeruginosa* is able to get energy from versatile respiratory mechanisms as well as from fermentative systems, which contribute to its ubiquitous distribution and persistence in diverse environments either under aerobic or anaerobic conditions. Therefore, we next wondered about the variability of functions controlled by Anr among different strains of *P. aeruginosa*. In addition to PAO1 strain, we analyzed the intraspecific diversity of the Anr regulon by exploring the set of genes controlled by Anr in seven *P. aeruginosa* strains from diverse origins: three isolated from environmental habitats (MTB-1, YL84, SJTD-1) and four clinical isolates from chronic cystic fibrosis (CF) lung infections (LESB58, DK2, RP73, SCV202065) (Supplementary Table S2).

Interestingly, the *in silico* analysis on the seven additional strains of *P. aeruginosa* did not render a significant increase in the size of Anr regulon observed in PAO1. Only 9 additional genes were incorporated to the whole *P. aeruginosa* pan regulon of 131 genes, 25 of which (20%) were absent or did not show the upstream Anr box in at least one of the analyzed genomes, indicating that the biological functions controlled by Anr are highly conserved among *P. aeruginosa* strains (Supplementary Table S2). Furthermore, no differential patterns were observed in relation to the environmental origin of the strains. As expected, the previously identified interspecific set of core genes, including *fbp*, was highly conserved in all *P. aeruginosa* strains and entirely belonged to the core genome of this species (Supplementary Table S2). In addition, the core Anr regulon was also composed by other genes related to the metabolic response to low-oxygen conditions (*ccoN2*) and the cytochrome c peroxidase (*ccpR*), denitrification (*dnr, nirS, nirQ, norC, nosS, narK1, narX, narL*), arginine fermentation (*arcD*) and other fermentation pathways (*adhA*, PA2119 and *ackA*). Furthermore, genes involved in key adaptive strategies, such as biofilm formation (*pilG)*, fimbria production (*cgrA*), hydrogen cyanide production (*hcnA*) and biodegradation (*alkB*) were also included. Interestingly, the set of shared genes under the control of Anr include genes that were described to be regulated by quorum sensing^10^, such as *hsiA2* (type IV secretion locus), PA3913 (putative collagenase) and PA5232 (part of a putative ABC transporter), in addition to *hcnA*, *nosR*, *narK1* and *ccpR*.

Most of the genes that belong to the core Anr regulon are also part of the *P. aeruginosa* core genome (Supplementary Table S2). On the other hand, the accessory genome of *P. aeruginosa* includes genes with conserved putative regulatory Anr-domains as well as genes of unknown function, which showed high strain-to-strain variability with respect to the presence of Anr boxes (Supplementary Table S2). Among them, there was a small set of genes (9) which contained Anr-boxes and was not present in the genome of PAO1 (extraPAO1).

The survey of the 13 interspecific Anr-core genes in the 95 assembled genomes available in Pseudomonas DB showed that they were conserved in almost 100% among the *P. aeruginosa* strains (Supplementary Table S3). In contrast, the set of strain-variable extraPAO1 genes was present in less than 50% of the genomes (Supplementary Table S3).

## Discussion

*Pseudomonas* genus shows remarkable metabolic and physiological versatility, enabling colonization of diverse ecological niches. From human opportunistic pathogens, as *P. aeruginosa*, to *P. extremaustralis* an Antarctic bacterium with biotechnological interest, a wide range of cellular functions can be found in these species in response to oxygen variations. These functions include the presence of high oxygen affinity cytochromes, the utilization of nitrate as alternative electron acceptor and arginine and pyruvate fermentation^2,4,8,31^ that have been described as controlled by Anr. However, anaerobic metabolism is not conserved among *Pseudomonas* species, while *P. aeruginosa* PAO1 can carry out a complete denitrification process^32^, *P. extremaustralis* is only able to reduce nitrate to nitrite, and both species can perform arginine and pyruvate fermentation^16,25^. On the contrary, *P. putida* KT2440 is not able to use nitrate as electron acceptor and neither carry out pyruvate fermentation^33^. However, less experimental information is available for other *Pseudomonas* species. In this work, we found that less than 50% of the 33 analyzed *Pseudomonas* genomes presented genes encoding nitrate reduction enzymes while arginine fermentation appeared as a more extended metabolism among them. Cytochromes with different oxygen affinities and characteristics like *azurin* and *cioA*, among others, could be found in the majority of the species suggesting a wide battery of genes for oxygen utilization.

Current knowledge about the Anr role in *Pseudomonas* is mainly based on *P. aeruginosa* PAO1 physiology and in lesser extent in *P. extremaustralis* and *P. putida* KT2440. *P. aeruginosa* PAO1 possesses not only the Anr regulator but also Dnr, which is sensitive to NO and is mostly related to denitrification control^34^. Notably, the Anr-box and Dnr-box are indistinguishable^3^. Due to this observation our *in-silico* analysis did not differentiate these regulators. It is important to point out that in the non-denitrifier analysed species, this regulator seems to be absent when a BlastP analysis is carried out.

Results of this work show Anr expression at mRNA and protein level in five *Pseudomonas* species including *P. protegens* Pf-5 and *P. syringae* pv. *syringae* B728a in which was not previously studied and suggest that Anr not only control a set of genes of the core genome, but also elements of the accessory genome, encoding functions probably associated with adaptation of each species. Whole genome *in silico* prediction of metabolic pathways results indicate that primitive or more conserved functions that can be attributed to Anr regulon are those related to carbon central metabolism and the optimization of oxygen utilization (heme metabolism and different oxygen affinity cytochromes). Although Anr was first described controlling anaerobic related genes, these functions seem to belong to the accessory Anr regulon, since the genes encoding key enzymes of anaerobic functions were absent in the majority of the species.

This is particularly interesting for biotechnological applications. For example, *P. putida* KT2440 has emerged as a new engineerable bacterium that displays as advantages a great metabolic adaptability, high tolerance to stress and is generally recognized as safe (GRAS) microorganism^14,35^. The manipulation of central metabolism is a key step for the production of different metabolites of interest^35^, particularly in the context of oxygen supply. In this bacterium glycolysis is reported to proceed through ED pathway, particularly through a cycle including not only ED but also pentose phosphate pathway and gluconeogenic reactions from the upper glycolysis^36^. In this context, the presence of Anr-boxes detected in this work in *edd* and *gap* genes could be relevant for metabolic engineering. Moreover, *hexR* and *gntR* regulators for glucose usage^37,38^ also presented Anr-boxes. In addition, reactions involved in these central metabolic routes endow to *P. putida* KT2440 with high NADPH regeneration rates^36^, allowing this bacterium to cope with oxidative stress and making it a remarkable host for redox-intensive reactions^14^. The coordination of carbon usage with oxygen availability could improve the efficiency in metabolism and can be used for biotechnological purposes. It has been shown that this bacterium possesses, in addition to the originally described Anr, two proteins belong to the Fnr/Crp family which are less sensitive to oxygen^39^. The presence of at least three oxygen sensitive regulators in this aerobic bacterium suggests their importance for environmental adaptability, and for fast and efficient response to changes in oxygen availability.

**Figure 5:**
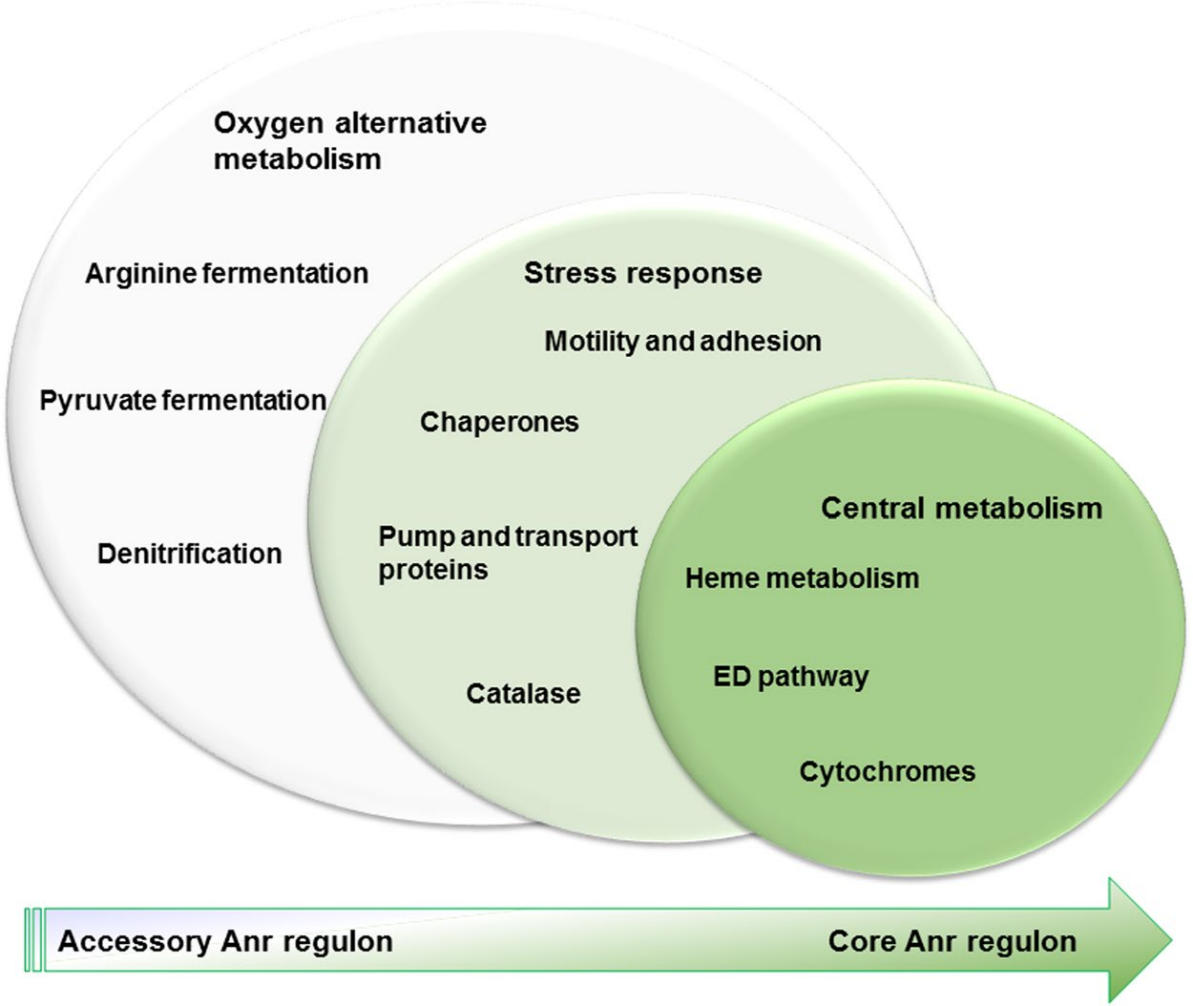
Proposed model showing conservation of functions controlled by Anr.

Soil presents oxygen gradients and microenvironments, which are variable according to distinct environmental conditions and/or bioactivities and interactions that are highly heterogeneous in the rhizosphere, whereas oxygen tension is low in soils with high humidity^40^. Thus, the control of several functions in plant-associated bacteria such as *P. protegens* Pf-5*, P. extremaustralis* or *P. syringae* B728a could be crucial for environmental survival. Indeed, biocontrol functions are regulated by Anr in different *Pseudomonas* species^18,41^ and in this work we detected Anr-boxes not only in genes related to central metabolism but also to other genes related with stress resistance and colonization (Fig. 5). These functions included some genes belonging to the core Anr regulon such as *cspD* encoding a cold shock protein, and the orthologous to PA2867 related to chemotaxis, besides others stress resistance associated genes like *katA*, *dnaJ*, *dnaK* and *groEL*. Genes related to motility that are also relevant for colonization such as *pilG*, encoding a component of Type IV pili implicated in cellular adhesion and *morA*, involved in signal transduction and flagella development, were detected. Decreased expression of both genes was observed in an *anr* mutant of *P. extremaustralis*^17^.

*P. aeruginosa* is a versatile opportunistic pathogen widely distributed, capable to respire oxygen as well as nitrogen oxides. Currently different reports indicate that its mode of respiration can be related to its distinct infection strategies, linking aerobic respiration to acute infections and microaerobic and anaerobic respiration to chronic infections mediated by biofilm mode of growth^42,43^. Particularly in the cystic fibrosis context, *P. aeruginosa* growths in oxygen restricted environments imposed by the thickened CF mucus, with local exacerbated hypoxia, and its anaerobic physiology is believed to play a key role in persistence functions^44–46^. Recently, it was reported that oxygen metabolism is a target in evolution of *P. aeruginosa* clinical clones^47^. In this sense, overexpression of denitrification and arginine fermentation, as well as certain high-affinity cytochrome oxidases genes^3^, indicate that Anr activity is high *in vivo*^48,49^. Moreover, several genes expressed under the control of Anr, like *azu*, cytochromes, *ccpR* and *icd* were reported as genetic markers for the metabolic adaptation to the CF lung environment, emphasizing the importance of oxygen metabolism during infection^50^. Our results are in line with these reports, since the Anr regulon is highly conserved among *P. aeruginosa* strains. The core Anr regulon was extensive and no major differences among the reference PAO1 strain, environmental and CF strains were observed, suggesting a pivotal role for Anr not only in the CF host but also in natural environments. Recently, examination of the quorum-sensing regulon from different free-living and host-associated *P. aeruginosa* strains revealed that quorum control of gene expression has a strain variable component, exhibiting the habitats from which strains were isolated^51^. On the contrary, we observed that genes included in Anr core regulon mainly belong to the core genome, supporting the view that Anr regulatory interactions appear to be mostly conserved. Thus, it seems that only a small strain-variable set of genes, related to accessory functions, would have been coupled to the Anr regulon during niche diversification among *P. aeruginosa* strains.

Thus, we proposed that the landscape of Anr regulon functions includes a set of more conserved functions such as those related to the central metabolism (core functions), others with an intermediate degree of conservation such as those related to stress responses and adaptability and finally, those that would be accessory, including anaerobic metabolism, which was surprisingly the first target described for Anr (Fig. 5).

Evolutionary expansion of the different species of *Pseudomonas* led to a great variety of the enzymes for energy metabolism. However, the regulatory machinery for energy metabolism based on Anr seems to be less variable, targeting key metabolic pathways that are common within the genus, inter and intraspecifically. The information provided in this study might be worth considering for exploring biotechnological innovations based on microbial metabolism.

## Methods

### Bacterial strains

The following *Pseudomonas* species were selected for analysis: *P. aeruginosa* PAO1^52^, *P. putida* KT2440^53^, *P. protegens* Pf-5^54^, *P. syringae* pv *syringae* B728a^55^ and, *P. extremaustralis* 14-3b^56^. Characteristics and genome accession number of the selected species are shown in Table 1.

### *In silico* analysis of Anr sequence

Anr nucleotide and protein sequences were obtained from public data bases (*anr* locus_ tag for *P. aeruginosa* PAO1: PA1544, *P. putida KT2440*: PP_4265, *P. protegens* Pf-5: PFL_1910, *P. syringae* pv.*syringae* B278a: Psyr_3425 and *P. extremaustralis* 14-3b: PE143B_0108070). Sequence were compared using Clustal Omega^57^.

### Phylogenetic analysis

Sequences were analyzed using the following programs available on line: BLAST (http://blast.ncbi.nlm.nih.gov/Blast.cgi), and bioinformatic tools included in the RAST server (http://rast.nmpdr.org/). The *Pseudomonas* genome database^58^ was also used to obtain comparative information. Phylogenetic analyses of proteins were performed using MEGA 5^59^. Phylogenetic trees were constructed using the neighbor-joining (NJ) method bootstrap analysis of 500 replicates and root on midpoint. Nucleotide analysis was carried out for the 16S rRNA subunit gene sequence. Accession number: PA5369.5 (*P.aeruginosa* PAO1), PFL_0119 (*P.protegens* Pf-5), Psy_RNA52 (*P.syringae pv. syringae* B728a), PP_16SA (*P. putida* KT2440) and PE143B_0130605 (*P. extremaustralis*).

### *In silico* determination of the Anr regulon

Potential Anr regulated genes in the *Pseudomonas* species genomes were evaluated with PRODORIC software using the *P. aeruginosa* Anr-Dnr box and the Fnr-box of *Escherichia coli* K12 matrix^60^. This search criterion was chosen because Anr and Dnr regulatory proteins of *P. aeruginosa* share an indistinguishable DNA consensus binding site^3^ and Anr is able to recognize Fnr binding sites^4^. For this reason, Anr, Dnr and Fnr consensus box sequences were included in this analysis. This approach, that involved the combination of several matrices included in PRODORIC, assigned a different score to each base position. All matrices used are very conservative in the bases located at both extremes of the box (4 bases each side) that are coincident in Anr and Dnr, and also for the homolog regulator Fnr, but allowed certain degree of flexibilization in the middle region of the box that has less conservation. The genome sequences of *P. aeruginosa* PAO1, *P. putida* KT2440, *P. protegens* Pf-5 and *P. syringae* pv. *syringae* B728a, available in the PRODORIC server were used, while for *P. extremaustralis* a manual genome search was performed. Only putative Anr-boxes located in intergenic zones up to 300 pb upstream of the ATG of the gene target were used for further analysis. Venn diagrams were constructed using InteractiVenn^61^. Manual analysis was also performed with selected genes in other representative *Pseudomonas* species. In case that the identity of the gene was not annotated, a BLAST analysis was carried out to compare with public data bases. Sequence of genes of other *Pseudomonas* species were obtained from *Pseudomonas* database^58^.

For *P. aeruginosa* Anr pan regulon determination, the presence of Anr-boxes was predicted by using the “Search DNA motif” tool from the *Pseudomonas* Genome DB (http://www.pseudomonas.com) and the Anr 5′-TTGATNNNNATCAA-3′-motif previously established^3^. Whole genomes sequences from seven *P. aeruginosa* strains were used: PACS2, AES-1R, FRD1, RP73, c7447m, DK2, LESB58, SCV20265 all of them available in *Pseudomonas* Genome DB.

Intergenic regions containing Anr-boxes in orthologous genes that were not present in PAO1 regulon (extraPAO1 regions) were determined by using the same procedure mentioned before and the presence of the Anr box was confirmed by PRODORIC using the Anr-Dnr-40 matrix^3^. In order to assess the distribution of these extraPAO1 regions in other *P. aeruginosa* strains, Blastn alignments of upstream regions were performed against the 95 complete genome sequences that are deposited at the *Pseudomonas* Genome DB. In order to compare the distribution of the Anr core/accesory regulon, this extended analysis was also performed with the 13 genes included in the Anr core regulon obtained from the interspecies analysis.

Information on whether Anr-controlled genes belong to the core or accessory *P. aeruginosa* genome was assessed through the BACTOME database, which includes 101 genomes (99 clinical isolates, and reference strains PAO1 and UCBPP PA14)^62^. Genes with >90% homology which are present in all *P. aeruginosa* isolates are considered to be part of the core genome.

### Growth conditions

*Pseudomonas* cultures were performed under microaerobic conditions in LB medium and LB supplemented with KNO_3_. Microaerobic cultures were performed using 50 ml of medium in 100-ml hermetically sealed bottles incubated at 30 °C and 50 rpm and harvested after 30 h of growth to ensure low oxygen conditions^4^.

### Quantitative real-time reverse-transcriptase PCR (qRT-PCR) experiments

Bacterial pellets of 5 ml of microaerobic cultures in LB medium supplemented with KNO_3_ were used for total RNA extraction using Total RNA Extraction Kit (RBC Biosciences). After treatment with DNaseI, cDNA was obtained using random hexamers (Promega) and Revert Aid Reverse Transcriptase (ThermoFisher Scientific, Waltham, USA) following the manufacturer’s instructions. qRT PCR was performed using a MyiQ2 Real-Time PCR Detection System (Bio-Rad Laboratories, Hercules, USA) and Real Time PCR mix (EvaGreen qPCR Mix Plus, no ROX, Solis Biodyne). The cycling conditions were as follows: denaturation at 95 °C for 5 min, 40 cycles at 95 °C for 25 s, 58 °C for 15 s, and 72 °C for 15 s, with fluorescence acquisition at 80 °C in single mode. For normalization 16S rRNA gene was used and relative changes in the expression of *anr* gene for microaerobic conditions was obtained through the relative standard curve method. Oligonucleotides used are detailed in Table S4.

### Anr antibody development and Western Blot assays

Antibody against native Anr was developed by a rational design. Anr sequences were aligned using ClustalO. Exposed amino acids corresponding to a functional domain were identified by comparing with Fnr from *Escherichia coli* which structure is available in public database. An immunogenic peptide was chosen by taking into account the conservation in *Pseudomonas* species and its immunogenic characteristics. The immunogenic peptide was specifically designed to do not recognize Dnr protein from *Pseudomonas* species. The designed peptide was synthesized and the polyclonal antibody was produced in rabbit by Abmart (Berkeley, USA). Then, the antibody was tested using *P. extremaustralis* total proteins to ensure its specificity and sensibility. Western Blot analysis was performed to detect Anr in the *Pseudomonas* spp. Aliquots of 1.5 ml of cultures grown under microaerobic conditions in LB medium supplemented with KNO_3_ were centrifuged at 12,000 rpm for 2 min. Pellets were resuspended in 1 ml PBS buffer with 1 mM PMSF and sonicated (Cole Parmer, ultrasonic homogenizer 4710) on ice. The lysates were centrifuged for 2 min at 12,000 rpm and the supernatants were transferred to a new tube. Total proteins in the supernatant were quantified by Bradford method. Aliquots containing 50 µg of proteins were electrophoresed using standard protocols in 4–12% polyacrylamide gel and then transferred to a PDVF membrane using a Semi-dry transfer system (TE 70 Amersham). Membranes were blocked overnight at 8 °C in a solution containing 0.02 M Tris-HCl, 0.5 M NaCl (pH 7.5), 0.05% v/v Tween-20 (TBST) and 5% w/v skimmed milk. The membrane was incubated with the rabbit polyclonal anti-Anr for 1.5 h and afterwards with anti-rabbit horseradish peroxidase-conjugated secondary antibody (1:5000, Santa Cruz Biotechnology) for 1.5 h. The resulting blots were incubated with enhanced chemiluminescence (ECL) reagent (GE Healthcare) and detected using Amersham imager 600 equipment. Negative controls were performed using an *anr* mutant of *P. extremaustralis*^16^ and positive controls using purified Anr protein of *P. extremaustralis*.

### Construction of the whole genome in silico metabolic network of *Pseudomonas* strains

To build the *in silico* metabolic networks (MN) of *P. extremaustralis* genome, we used the PathoLogic module of Pathway Tools v. 18.0^63^. PathoLogic creates a Pathway/Genome Database (PGDB) containing the predicted metabolic pathways of a given organism using as input an annotated genome in GenBank format. Metabolic reconstruction includes determining gene-protein-reaction-pathway associations, which are primarily based on the corresponding enzyme commission (EC) number. After automatic reconstruction, manual curation of pathways of interest was performed (tetrapyrrole biosynthesis I (from glutamate), tetrapyrrole biosynthesis II (from glycine), superpathway of heme biosynthesis from uroporphyrinogen-III, heme biosynthesis II (anaerobic), basically by filling of enzymatic “holes”, reactions in metabolic pathways for which no enzyme is identified, by looking for functional orthologs using BLAST. Afterwards, the metabolic network was exported in systems biology mark-up language (SBML) format for downstream analyses. Reactions involving only small-molecules were considered. After exporting MN construction, we filtered potential currency compound^64,65^. Cytoscape v. 3.1.0^66^ was then used for network visualization and calculation of topological metrics. In this representation, nodes represent reactions, and there exists an edge between two nodes if a product of a reaction is used as substrate on the reaction that follows. Betweenness centrality (BC) was also calculated for each node in MN. The size of the nodes on the graph representation is proportional to their centrality value. High values of Betweenness centrality for a node indicate its participation as an important communication path, bridging different metabolic parts^67^.

### Statistical analysis

Differences between means were determined through one-way analysis of variance (ANOVA) and t-test, with a P < 0.05 significance level.

## Supplementary information

Supplementary Figures

Table S1

Table S2

Table S3

Table S4
