# Supplementary material for: Core regulon of the global anaerobic regulator Anr targets central metabolism functions in Pseudomonas species
Source: Sci Rep. 2019 Jun 21;9:9065. doi: 10.1038/s41598-019-45541-0 (PMC6588701; doi:10.1038/s41598-019-45541-0)
Supplement: Supplementary file 1 — Supplementary Figures [file 41598_2019_45541_MOESM1_ESM.pdf]

Core regulon of the global anaerobic regulator Anr targets central metabolism functions in *Pseudomonas* species

Paula M. Tribelli[1,2], Adela M. Lujan[3,4], Agustín Pardo[1], José G. Ibarra[1], Darío Fernández Do Porto[5], Andrea Smania [3,4], Nancy I. López[1,2*]

[1]IQUIBICEN, CONICET, [2]Departamento de Química Biológica, Facultad de Ciencias Exactas y Naturales, Universidad de Buenos Aires, Argentina [3]Universidad Nacional de Córdoba. Facultad de Ciencias Químicas, Departamento de Química Biológica Ranwel Caputto. Córdoba, Argentina. [4]CONICET, Centro de Investigaciones en Química Biológica de Córdoba (CIQUIBIC), Córdoba, Argentina, [5]Instituto de Cálculo, Facultad de Ciencias Exactas y Naturales, UBA, Buenos Aires, Argentina.

*Address correspondence to Nancy I. López, nan@qb.fcen.uba.ar

Figure S1

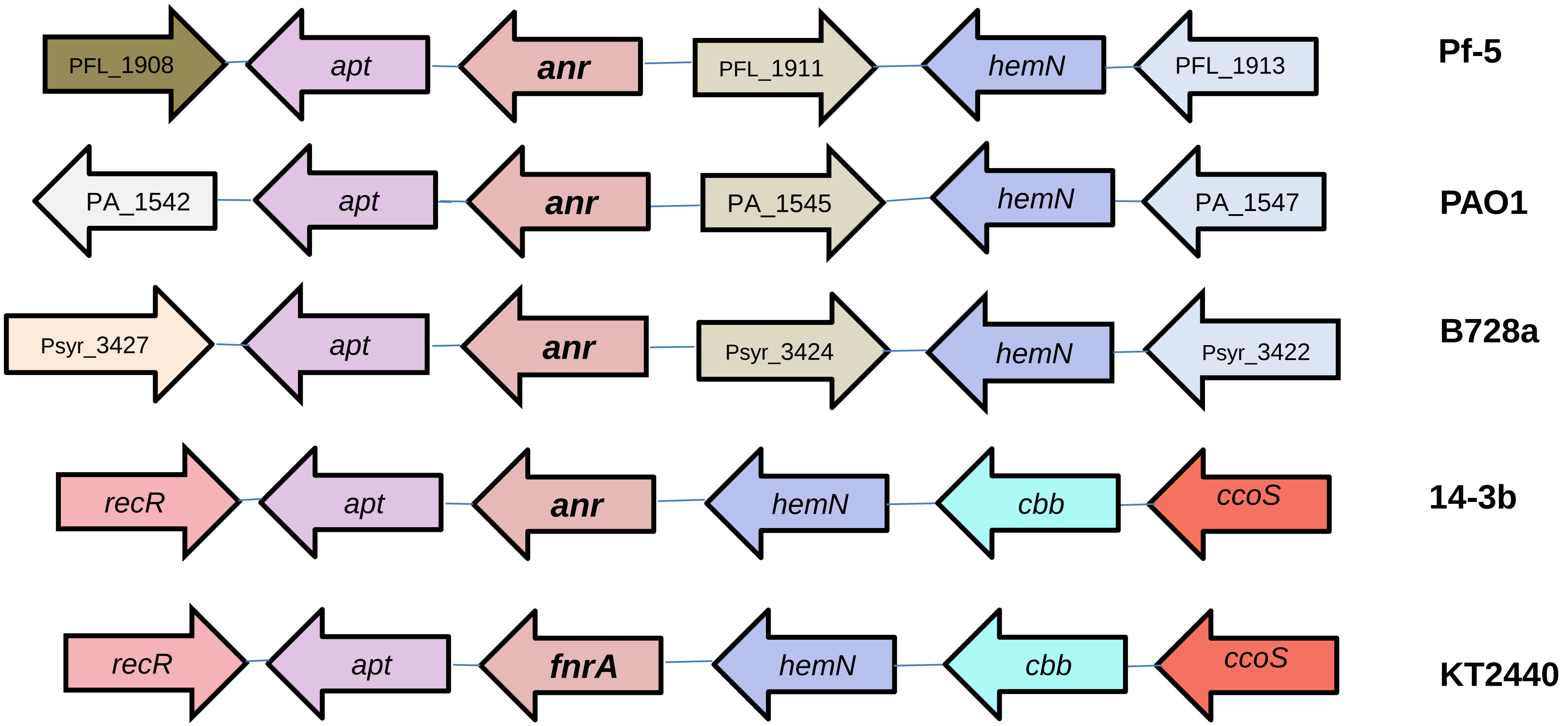

Figure S1. Genomic region showing anr location in the different Pseudomonas spp. In P. putida KT2440 this gene is annotated as fnrA. Same colour denotes orthologous genes. Genes encoding hypothetical proteins are shown in grey. P. aeruginosa PAO1 (PAO1); P. protegens Pf-5 (Pf-5); P. putida KT2440 (KT2440), P.extremaustralis 14-3b (14-3b) and P.syringae pv. syringae B728a (B728a).

Figure S2

```
P.ae        MAETIKVRALPQAHCKDCSLAPLCLPLSLTVEDMDSLDEIVKRGRPLKKGEFLFRQGDPF
P.sy        MSEPVKLRAQTQAHCKDCSLAPLCLPLSLNTEDMDCLDQIVKRGRPLKKGEFLFRQGDTF
P.put       MSEPVKLRPHNQAHCKDCSLAPLCLPLSLNLEDMDALDEIVKRGRPLKKGEFLFRQGDSF
P.prot      MSEPVKLRAHNQAHCKDCSLAPLCLPLSLNLEDMDALDEIVKRGRPLKKGEFLFRQGDGF
P.ext       MSEPVKLRAHSQAHCKDCSLAPLCLPLSLNLEDMDALDEIVKRGRPLKKGEFLFRQGDKF
            *:* :*:*   ******************. ****.**:******************* *

P.ae        GSVFAVRSGALKTFSITDAGEEQITGFHLPSELVGLSGMDTETYPVSAQALETTSVCEIP
P.sy        ESVYAVRSGALKTFNISDSGEEQLTGFHLPSELVGMSGMDAEAYPVSAQALETTSVCEIP
P.put       GSVYAVRSGALKTFSLSDSGEEQITGFHLPSELVGLSGMDTEAYPVSAQAQETTSVCEIP
P.prot      DSVYAVRSGALKTFSLSDSGEEQITGFHLPSELVGLSGMDTESHPVSAQALETTSVCEIP
P.ext       DCVFAVRSGALKTFSLSDGGEEQITGFHLPSELVGLSGMDTEMHPVSAQALETTSVCEIP
             .*:**********.::*.****:***********:****:* :****** *********

P.ae        FERLDELSEQLPQLRRQLMRLMSREIRDDQQMMLLLSKKTADERIATFLVNLSARFRARG
P.sy        FERLDELSVRLPQLRRQLMRVMSREIRDDQQMMLLLSKKTADERIATFLINLSARFRARG
P.put       FERLDELSVQLPQLRRQLMRVMSREIRDDQQMMLLLSKKTADERIATFLVNLSARFRARG
P.prot      FERLDELALQLPQLRRQLMRVMSREIRDDQQMMLLLSKKTADERIATFLVNLSARFRARG
P.ext       FERLDELALQLPQLRRQLMRVMSREIRDDQQMMLLLSKKTADERIATFLVNLSARFRARG
            *******: :**********:****************************:**********

P.ae        FSAQQFRLAMSRNEIGNYLGLAVETVSRVFTRFQQNGLISAEGKEVHILDSIELCALAGG
P.sy        FSANQFRLSMSRNEIGNHLGLAVETVSRVFTRFQQNQLISAEGKEIHILDPIELCALAGG
P.put       YSANQFRLSMSRNEIGNYLGLAVETVSRVFTRFQQNGLLRAEGKEVHILDPIQLCALAGG
P.prot      FSANQFRLSMSRNEIGNYLGLAVETVSRVFTRFQQNELIAAEGKEVHILDPIQLCALAGG
P.ext       FSANQFRLSMSRNEIGNYLGLAVETVSRVFTRFQQNELIAAEGKEVHILDPIQLCALAGG
            :**:****:********:****************** *: *****:**** *:*******

P.ae        QLEG-
P.sy        SMQS-
P.put       AIEA-
P.prot      SVEG-
P.ext       SLEGQ
             ::.
```

Figure S2. Alignment of Anr sequence of different *Pseudomonas* species. Essential residues for its function are marked. Cysteines bind to Fe-S cluster sensitive to oxygen are shown in green color and in red key residues for DNA binding. The HTH domain is underlined.

Figure S3

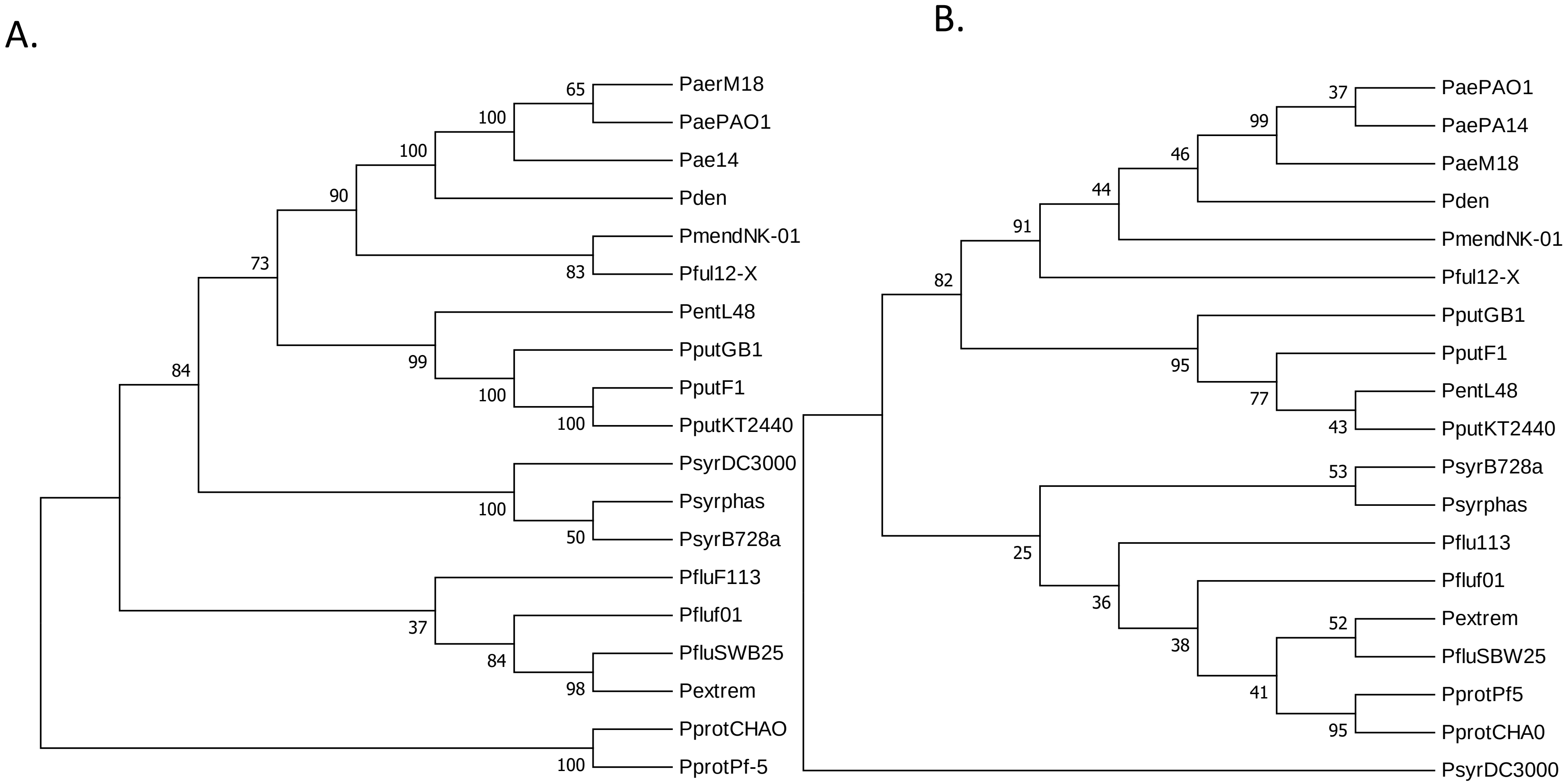

Figure S3. Evolutionary relationships of *Pseudomonas* spp. based on Neighbor-Joining method of *anr* and 16S rRNA genes. A. *anr*. B. 16S rRNA gene. Pae.: *P. aeruginosa*: M18, PAO1, PA14, Pden: *P. denitrificans*, PmendNK-01: *P. mendocina* NK-01, Pful12-X: *P.fulva*12-X, PentL48: *P.entomophila* L48, Pput.: *P. putida* GB-1, F1, KT2440, Pprot: *P.protegens* CHA01, Pf-5, Pflu: *P.fluorescens* F113, Pf01, SWB25, Pextrem: *P.extremaustralis* 14-3b, PsyrDC3000: *P.syringae pv. tomato* DC3000, Psyphas: *P.syringae pv. phasicola*, PsyrB278: *P.syringae pv. syringae* B728a. Bootstrap test 500 replicates.

Figure S4

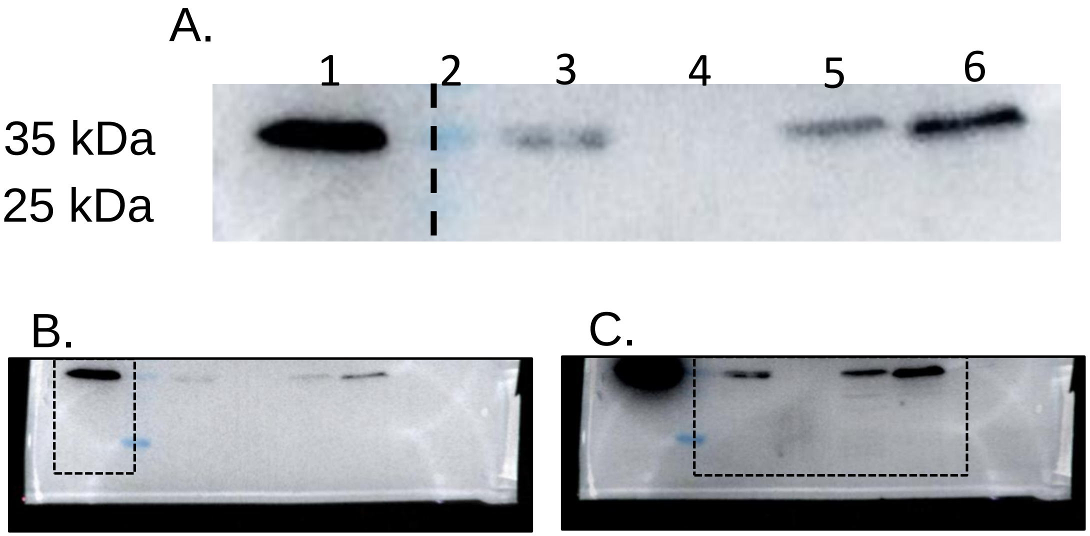

Figure S4: Western Blot analysis of Anr. Microaerobic cultures were carried out in sealed bottles with 1:2 medium to flask volume ratio and low agitation (50 rpm) incubated for 30h. Cultures were performed in LB medium supplemented with KNO_3. A: Merged image of panels B and C, dashed line indicates the merging point. Lines: 1. Purified recombinant Anr protein of *P. extremaustralis* used as positive control 2: Marker PageRuler prestained protein ladder (Thermo scientific); 3: *P. extremaustralis*; 4: anr mutant of *P. extremaustralis*16 used as negative control. 5: *P. syringae* B728a: 6: *P. putida* KT2440. Panels B and C corresponds to the original pictures used to build panel A, images were taken from the same Western-blot assay with different exposure time: 10 seconds and 1 minute, respectively. Dashed lines indicates the cropped site used to build panel A.

Figure S5

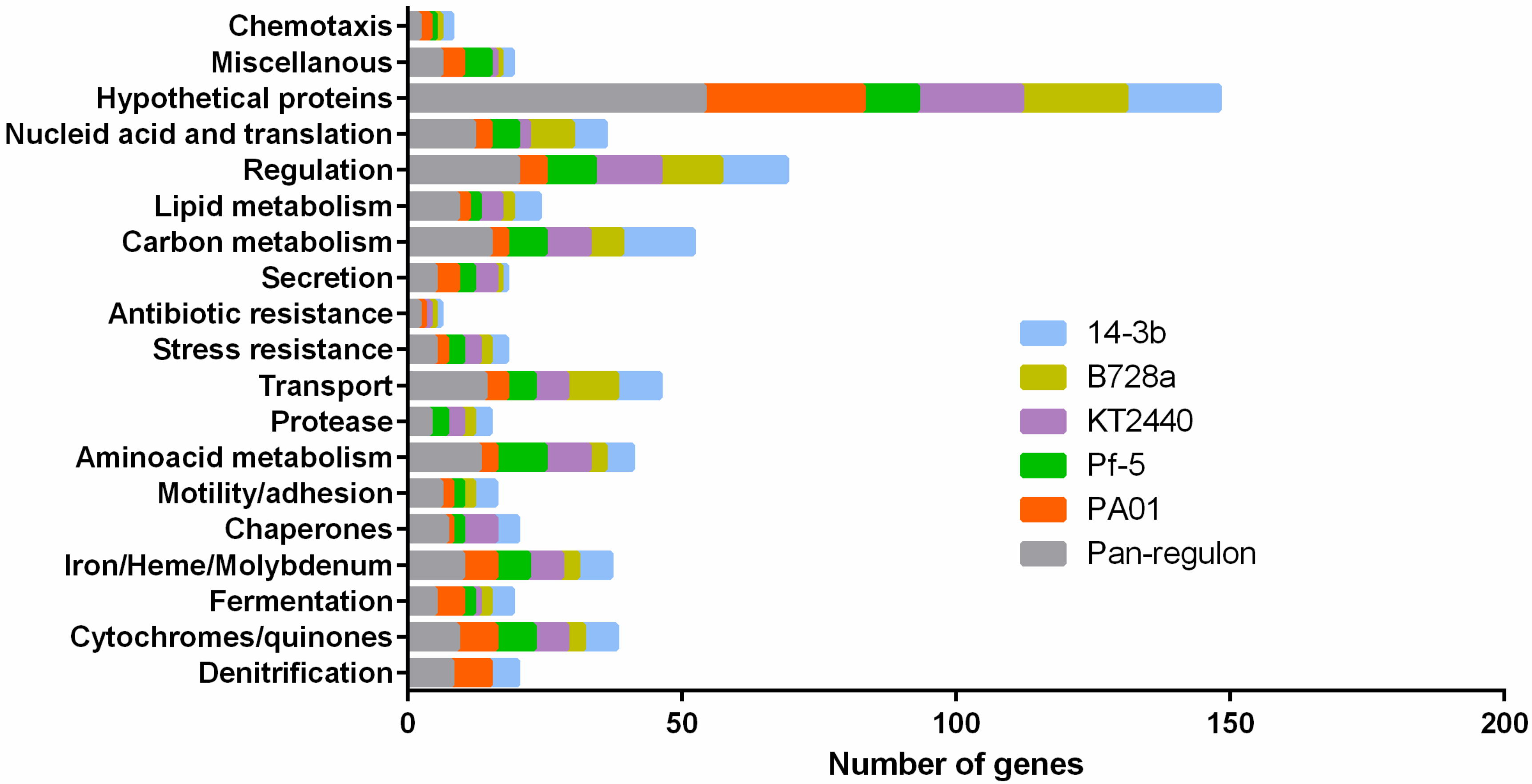

Figure S5. Functional classification of the genes presenting an Anr-box in its promotor zone. Fragments of different colors in the bars represent the number of Anr box containing genes in each functional category for *P. aeruginosa* PAO1 (PAO1); *P. protegens* Pf-5 (Pf-5); *P. putida* KT2440 (KT2440), *P. extremaustralis* 14-3b (14-3b) and *P. syringae* pv. *syringae* B728a (B728a). In each functional category, the number of genes belonging to the pan regulon, the entire Anr-box containing gene set of all species, is shown in grey color. Pan regulon includes genes with Anr-box present in all species (core regulon) and genes present only in some species (variable or accessory regulon).

Figure S6

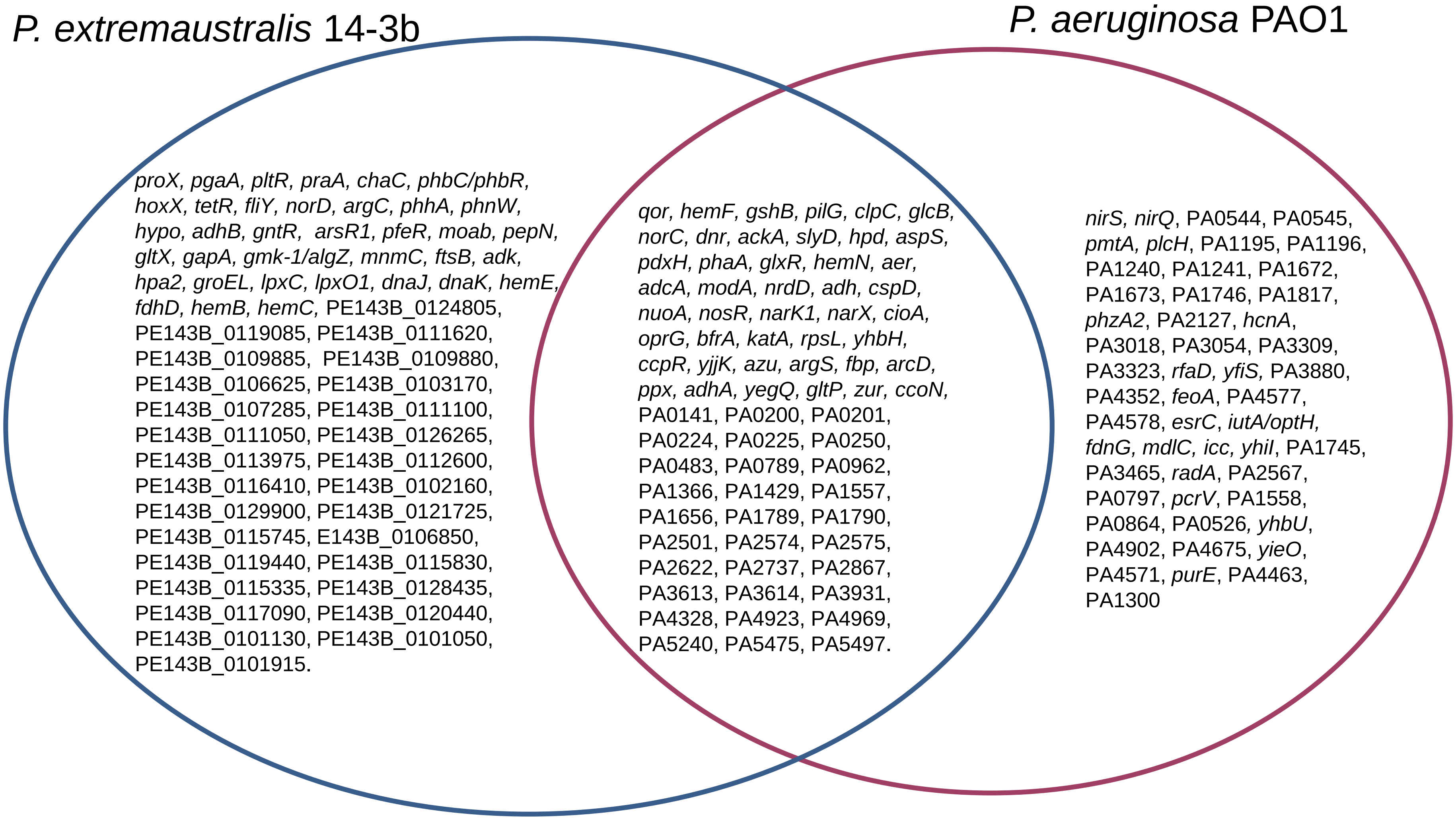

Figure S6. Genes with Anr-box in the promotor zone present in *P.aeruginosa* PAO1 and *P.extremaustralis* 14-3b.

Figure S7

Taxonomical group or subgroup	*Pseudomonas* spp.	*narG*	*arcB*
***P. fluorescens* subgroup**	*P. fluorescens* SBW25	NP	
	P. fluorescens WH6		
	P. extremaustralis 14-3b		
***P. gessarii* subgroup**	*Pseudomonas* sp. Ag1	NP	
	Pseudomonas sp. PAMC 25886	NP	
***P. fragii* subgroup**	*P. psychrophila* HA-4	NP	
***P. jessenii* subgroup**	*Pseudomonas* sp. GM74	NP	
	Pseudomonas sp. UW4	NP	
***P. koreensis* subgroup**	*P. fluorescens* Pf01	NP	
	Pseudomonas sp. GM30	NP	
***P. mandeleii* subgroup**	*P. mandelii* JR-1		
	Pseudomonas sp. GM50	NP	
***P. chlororaphis* subgroup**	*P. protegens* Pf-5	NP	
	P. chlororaphis O6		
***P. corrugata* subgroup**	*P. brassicacearum sub. brassicacearum*		
	P. mediterranea CFBP 5447		
***P. syringae* subgroup**	*P. syringae pv. tomato* DC3000	NP	
	P. syringae B728a	NP	
***P. putida* subgroup**	*P. putida* KT2440	NP	
	P. putida BIRD-1	NP	
	P. putida F1	NP	
***P. straminea* group**	*P. fulva* 12-X	NP	NP
***P. aeruginosa* group**	*P. aeruginosa* PAO1		
	P. aeruginosa PA14		
***P. oleovorans* group**	*P. mendocina* DLHK	NP	
	P. mendocina NK-1	NP	
	P. oleovorans MOIL14HWK12	NP	NP
***P. orzihabitants* group**	*P. psychrotolerans L19*	NP	NP
***P. stutzeri* group**	*P. stutzeri* A1501		
	P. stutzeri DSM 4166		
	P. stutzeri T13		
	P. stutzeri NF13	NP	NP
	P. stutzeri RCH2		

Figure S7: Presence of *narG* and *arcB* in *Pseudomonas* spp. Green: gene present; NP: not present
