# Supplementary material for: Core regulon of the global anaerobic regulator Anr targets central metabolism functions in Pseudomonas species
Source: Sci Rep. 2019 Jun 21;9:9065. doi: 10.1038/s41598-019-45541-0 (PMC6588701; doi:10.1038/s41598-019-45541-0)
Supplement: Supplementary file 5 — Table S4 [file 41598_2019_45541_MOESM5_ESM.docx]

**Core regulon of the global anaerobic regulator Anr targets central metabolism functions in *Pseudomonas*** **species**

Paula M. Tribelli^1,2^, Adela M. Lujan^3,4^, Agustín Pardo^1^, José G. Ibarra^1^, Darío Fernández Do Porto^5^, Andrea Smania ^3,4^, Nancy I. López^1,2*^

^1^IQUIBICEN, CONICET, ^2^Departamento de Química Biológica, Facultad de Ciencias Exactas y Naturales, Universidad de Buenos Aires, Argentina ^3^Universidad Nacional de Córdoba. Facultad de Ciencias Químicas, Departamento de Química Biológica Ranwel Caputto. Córdoba, Argentina. ^4^CONICET, Centro de Investigaciones en Química Biológica de Córdoba (CIQUIBIC), Córdoba, Argentina, ^5^Instituto de Cálculo, Facultad de Ciencias Exactas y Naturales, UBA, Buenos Aires, Argentina.

*Address correspondence to Nancy I. López, nan@qb.fcen.uba.ar

Table S4. Oligonucleotides used for qRT PCR assays

| Name | Sequence | Gene | Species |
| --- | --- | --- | --- |
| Anr1 forward | GCCCAGGCCCTGGAAAC | *anr* | *P.aeruginosa* PAO1 and *P.extremaustralis* |
| Anr1 reverse | ATCATCTGCTGGTCATCGC | *anr* | *P.aeruginosa* PAO1 and *P.extremaustralis* |
| Anr2 forward | TGAACAAGCCGCTG | *anr* | *P.putida* KT2440, *P.syringae* pv*.syringae* B728a and *P.protegens* Pf-5. |
| Anr2 reverse | ACCGAGTCGCCTTC | *anr* | *P.putida* KT2440, *P.syringae* pv.*syringae* B728a and *P.protegens* Pf-5. |
| 16S1 forward | AGCTTGCTCCTTGATTCAGC | r16RNA |  |
| 16S1 reverse | AAGGGCCATGATGACTTGAC | r16RNA |  |
